# Discussion of hemodynamic optimization strategies and the canonical understanding of hemodynamics during biventricular mechanical support in cardiogenic shock: does the flow balance make the difference?

**DOI:** 10.1007/s00392-024-02377-7

**Published:** 2024-01-23

**Authors:** Nikolaos Patsalis, Julian Kreutz, Giorgos Chatzis, Carlo-Federico Fichera, Styliani Syntila, Maryana Choukeir, Sebastian Griewing, Bernhard Schieffer, Birgit Markus

**Affiliations:** Department of Cardiology, Angiology, and Intensive Care Medicine, University Hospital of the Philipps University of Marburg, Baldinger Str., 35043 Marburg, Germany

**Keywords:** Cardiogenic shock, Mechanical circulatory support, Hemodynamic monitoring, Organ perfusion

## Abstract

**Background:**

Mechanical circulatory support (MCS) devices may stabilize patients with severe cardiogenic shock (CS) following myocardial infarction (MI). However, the canonical understanding of hemodynamics related to the determination of the native cardiac output (CO) does not explain or support the understanding of combined left and right MCS. To ensure the most optimal therapy control, the current principles of hemodynamic measurements during biventricular support should be re-evaluated.

**Methods:**

Here we report a protocol of hemodynamic optimization strategy during biventricular MCS (VA-ECMO and left ventricular Impella) in a case series of 10 consecutive patients with severe cardiogenic shock complicating myocardial infarction. During the protocol, the flow rates of both devices were switched in opposing directions (+ / − 0.7 l/min) for specified times. To address the limitations of existing hemodynamic measurement strategies during biventricular support, different measurement techniques (thermodilution, Fick principle, mixed venous oxygen saturation) were performed by pulmonary artery catheterization. Additionally, Doppler ultrasound was performed to determine the renal resistive index (RRI) as an indicator of renal perfusion.

**Results:**

The comparison between condition 1 (ECMO flow > Impella flow) and condition 2 (Impella flow > VA-ECMO flow) revealed significant changes in hemodynamics. In detail, compared to condition 1, condition 2 results in a significant increase in cardiac output (3.86 ± 1.11 vs. 5.44 ± 1.13 l/min, *p* = 0.005) and cardiac index (2.04 ± 0.64 vs. 2.85 ± 0.69, *p* = 0.013), and mixed venous oxygen saturation (56.44 ± 6.97% vs. 62.02 ± 5.64% *p* = 0.049), whereas systemic vascular resistance decreased from 1618 ± 337 to 1086 ± 306 s*cm^−5^ (*p* = 0.002). Similarly, RRI decreased in condition 2 (0.662 ± 0.05 vs. 0.578 ± 0.06, *p* = 0.003).

**Conclusions:**

To monitor and optimize MCS in CS, PA catheterization for hemodynamic measurement is applicable. Higher Impella flow is superior to higher VA-ECMO flow resulting in a more profound increase in CO with subsequent improvement of organ perfusion.

**Graphical Abstract:**

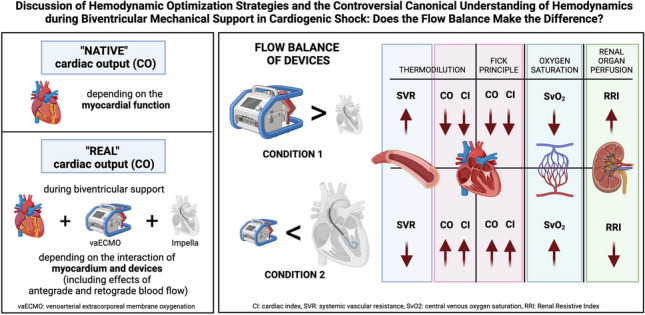

## Introduction

Cardiogenic shock (CS) is a condition of severe myocardial and circulatory failure. To stabilize hemodynamics in CS, extracorporeal mechanical circulatory support (MCS), i.e., veno-arterial membrane oxygenation (VA-ECMO) and Impella (Abiomed, Danvers, MA) have become established therapeutic strategies during the last years [1–3]. However, despite the use of innovative therapeutic regimes including MCS, the total 6–12 months mortality of CS remains higher than 50% without any noteworthy improvement in the last two decades [4]. Thus, it becomes evident that the implantation of MCS alone does not necessarily guarantee the success of therapy subsequently improving patients’ prognosis. Both the understanding of the complex physiology of hemodynamic processes during mechanical circulatory support and the definition of therapeutic strategies in everyday clinical practice seem to be essential for more adequate therapy control.

Over the past decade, we learned that, in CS patients treated with VA-ECMO, the implementation of an additional left ventricular unloading with Impella (so-called ECMELLA) is associated with a lower mortality rate in comparison to VA-ECMO alone [5–7]. Thereby, Impella not only increases cardiac index (CI) and cardiac output (CO) but also reduces systemic vascular resistance (SVR), and serum lactate as indicators for improved tissue and organ perfusion [8, 9].

The most optimal flow balance however of opposing laminar blood flows in biventricular support remains to be elucidated. In principle, for adequate therapy control in this complex situation of counteracting MCS, invasive hemodynamic monitoring is required [10, 11]. The canonical understanding of invasive hemodynamic measurement techniques related to the determination of exclusively native CO points to the limitations of this technique during biventricular support. Since historical studies are based on the measurement of native cardiac output (without MCS), this CO calculation cannot be easily transferred to the biventricular setting in which counteracting blood flows of both devices together with the drainage of added fluids affect the accuracy of invasive measurement of the native CO [12–14]. Thus, the patient’s real cardiac output is the sum of both counteracting devices plus the remaining native myocardial contractility. In this regard, established invasive hemodynamic parameters should be interpreted differently for therapy control during biventricular support.

Therefore, we here engage the discussion on the optimized flow balance of MCS devices and options for hemodynamic measurement including their limitations during VA-ECMO and Impella therapy in CS. We further addressed the question about the complexity of invasive hemodynamic measurements during biventricular support, comparing different and independent measurement techniques and parameters (thermodilution, Fick principle, mixed venous oxygen saturation, and Doppler ultrasound to determine the renal resistive index (RRI)) to validate hemodynamic parameters of the closed circuit of “myocardium-VA-ECMO-Impella”-system. Thus, to better represent the peculiarities of hemodynamics and validate our discussion, we here compared the impact of pre-defined flow levels of opposing VA-ECMO and Impella support on hemodynamics, vascular resistance, and renal organ perfusion in patients with CS-complicated myocardial infarction.

## Methods

### Study population

**Fig. 1:**
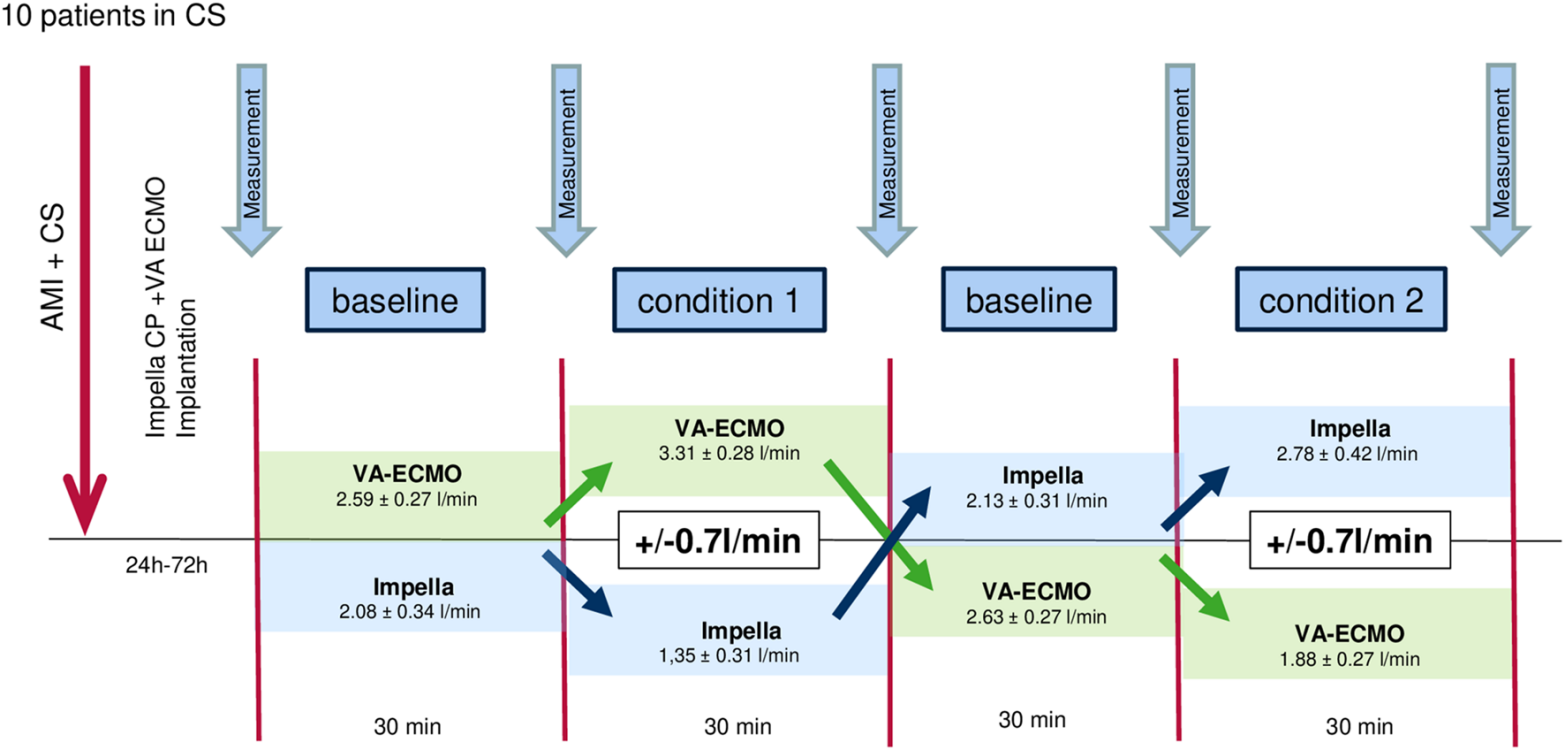
Measurements of hemodynamic parameters according to a standardized protocol during ECMELLA therapy. The parameters documented for each measurement were the BP, MAP, catecholamine doses, RRI (right/left, 3 times), CI, CO, and SVR. AMI, acute myocardial infarction; BP, blood pressure; CS, cardiogenic shock; CO, cardiac output; CI, cardiac index; MAP, mean arterial pressure; SVR, systemic vascular resistance; RRI, renal resistive index

At the University Hospital of Marburg, from January 2021 to September 2022, 18 patients with refractory CS complicating myocardial infarction were treated with both VA-ECMO and Impella CP left ventricular microaxial pump. In 10 patients, who survived the acute phase of CS, in a stabilized hemodynamic situation, changes in hemodynamics and vascular resistance were documented during different flow settings of biventricular mechanical circulatory support. This was to define the most optimal flow balances of VA-ECMO and Impella (Fig. 1).

The presence of CS at the time point of device implantation was defined as systolic blood pressure below 90 mmHg over a period longer than 30 min or the need for vasopressor therapy to maintain systolic blood pressure higher than 90 mmHg. At least one or a combination of the following conditions had to be documented: impaired mental function, oliguria with urine output < 30 ml/h, cold and wet skin, and lactate in blood serum (> 2.0 mmol/l) [15].

In two patients, VA-ECMO was implanted as the first therapeutic strategy due to a mixed hemodynamic and respiratory failure on admission day. In these patients, the Impella was implanted on day 2 of therapy due to a left ventricular dilatation and further worsening of the hemodynamic situation. In another two patients, Impella implantation took place on admission and a day later, the VA-ECMO was implanted due to respiratory and hemodynamic insufficiency. The remaining six patients received the Impella and the VA-ECMO simultaneously. In all patients, a pulmonary artery catheter (PAC) was implanted in the cath lab according to the established shock algorithms.

### Invasive hemodynamic parameters

Different techniques for hemodynamic measurements were used to maintain the most optimal accuracy of the data. To evaluate hemodynamic changes, measurements of cardiac output (CO), cardiac index (CI), and systemic vascular resistance (SVR) first were performed based on the thermodilution principle by invasive pulmonary artery catheterization. To validate the results, CO and CI were additionally calculated by the Fick principle (CO = VO_2_/AVDO_2_). VO_2_ describes the estimated oxygen intake, and AVDO_2_ is the difference between arterial oxygen concentration and mixed venous oxygen saturation (SvO_2_). During the measurements which were conducted in a very short period of time (approximately 3 h), no changes of ventilator settings took place including the FiO_2_. O_2_ concentration of the VA-ECMO was constantly set at 50% and no significant alterations of paO_2_ (measured via right radial artery to avoid overestimated O_2_ measurements due to the “watershed phenomenon” of VA-ECMO support) were documented [16]. VO_2_ was estimated considering the body surface area (BSA), age, and sex of the patients using the following formulas: VO_2-male_ = BSA × (161 − age × 0.54), VO_2-female_ = BSA × (147.5 − age × 0.47). These formulas for estimating oxygen uptake apply to spontaneously breathing patients. The fact that the O_2_ concentration (mechanical ventilation and VA-ECMO) and paO_2_ remained unchanged suggests that the oxygen uptake of the patients also remained without significant changes during the measurements.

It should be emphasized again, that the goal of hemodynamic measurement during biventricular support was not to calculate the “native” CO, but the “real” CO and hemodynamic situation depending on the communication and interaction of the entire system of the patient and the machines.

### Mixed venous oxygen saturation (SvO_2_)

To confirm the results of the hemodynamics measurements in the best possible way, SvO_2_ as a parameter representing cardiac output was taken via pulmonary artery catheterization during each condition of flow change of the devices. SvO_2_ reflects the balance between global oxygen delivery (DO_2_) and oxygen consumption (VO_2_).

### Renal resistive index (RRI)

The RRI was routinely determined in all CS patients as an established indicator for the occurrence and reversibility of acute kidney injury (AKI) [17–20]. RRI was determined by intrarenal artery Doppler measurements (peak systolic velocity minus the end-diastolic velocity divided by the peak systolic velocity). Although many concomitant factors like sex, age, diabetes, peripheral and coronary artery disease, and vasculature stiffness as well as the application of vasopressors alter RRI, it correlates with renal vascular resistance and thus renal organ perfusion. RRI values between 0.6 and 0.7 constitute the normal range [21].

An elevated RRI (≥ 0.7) relates to an enhanced risk of acute kidney injury (AKI) and mortality. RRI levels higher than 0.8 are associated with an increased risk of need for renal replacement therapy (RRT) and chronic renal insufficiency in critically ill patients [22–25]. Our previous data indicate that the Impella improves RRI, indicating improved renal organ perfusion while increasing laminar blood flow [8, 26].

In every patient, the RRI was routinely obtained for both kidneys. RRI values between the left and right kidney showed a mean difference of 0.008 ± 0.01 (*p* = 0.16). There was no patient with a difference of more than 0.05.

### Study protocol

At baseline, before starting the measurements, all the patients showed stable hemodynamics. The MAD was about 87.55 ± 15.85 mmHg in the overall cohort, with accordingly very limited need for vasoconstrictor therapy (norepinephrine). The targeted MAD was between 60 and 70 mmHg.

As depicted in Fig. 1, all patients underwent the following procedure: A first measurement of hemodynamics and vascular resistances, including the RRI, occurred at a baseline output level (BL) of 2.59 ± 0.27 l/min of the VA-ECMO and 2.08 ± 0.32 l/min of the Impella. After 60 min, another “safety measurement” was performed at the same support level of the devices. The therapeutic regime remained without any changes during this period. This was to verify and ensure stable hemodynamics of the patients before variation of the output of the VA-ECMO and Impella took place to individually optimize the therapeutic strategy. Next, the VA-ECMO output was increased by a mean of 0.7 l/min (± 0.18 l/min) and the Impella performance level was reduced by a mean of 0.7 l/min (± 0.21 l/min). Thirty minutes later, the third measurement took place on this support level of the devices (condition 1). The following measurement, 30 min later, was on baseline settings again in order to get back to the starting conditions of the measurements. After that, the VA-ECMO output was decreased by a mean of 0.7 l/min (± 0.23 l/min) below baseline level and the Impella output was enhanced by a mean of 0.7 l/min (± 0.20 l/min) above baseline level (condition 2). Another 30 min later, the fifth measurement took place during this second condition (Fig. 1).

In order to be able to compare the flow settings of the MCS devices as validly as possible, we aimed for an equal variation of the performance levels of Impella and VA-ECMO. As known, the maximum performance of VA-ECMO and Impella is dependent and very often limited by several factors, such as hypovolemia or right ventricular dysfunction. During the protocol, a flow variation of a mean of 0.7 l/min of each, the VA-ECMO and Impella, meaning a total difference of ≈1.4 l/min between the devices could be safely implemented without disturbing the smooth function of both MCS devices. Furthermore, the duration of each flow setting and measurement at the end of each condition with 30 min on steady state was to ensure that hemodynamic changes could occur. The fact that the chosen time period was sufficient could be validated in the context of recurrent baseline measurements, which showed comparable results without significant differences between the measurements.

During the measurements, dosages of catecholamines and fluids, setting of invasive mechanical ventilation (pressure-controlled), and right and left ventricular ejection fraction remained without any changes. Furthermore, the heart rate, the mean arterial pressure, and central venous pressure of the patients did not show any noteworthy alterations while changing the flow levels of the devices.

### Clinical data/parameters

Further treatment-relevant variables were documented as follows: respirator parameters of mechanically ventilated patients, volume substitution throughout the measurements, heart rate, arterial blood pressure including systolic, diastolic mean arterial pressure, and catecholamine dosages. In addition, routine blood parameters including GFR and the need for renal replacement therapy (RRT) were registered.

### Statistical analysis

Data were presented as absolute variables and percentages for categorical variables and as mean with ± standard deviation for continuous variables. The Shapiro–Wilk and the Pearson tests were implemented for examining for normal distribution and subsequently, univariate ANOVA was conducted to evaluate for differences among the various conditions. The calculation of intraobserver variability was based on the ICC and its 95% CI.

For visualization, all analyses were performed with SPSS 27 (IBM, New York, NY) and GraphPad Prism 7.0 (GraphPad Software, San Diego, CA). A *p*-value less than 0.05 was considered statistically significant.

## Results

Hemodynamic data of 10 patients with cardiogenic shock (CS) complicating myocardial infarction supported with VA-ECMO and Impella (ECMELLA) were analyzed for further discussion of therapy optimizing strategies during biventricular support. The baseline characteristics of the patients and demographics are summarized in Table 1.

**Table 1:** Demographics and baseline characteristics

Patient characteristics	
Age (years)	67 ± 7.6
Female (%)	20
Male (%)	80
1-vessel CHD (*n*)	3
2-vessel CHD (*n*)	3
3-vessel-CHD (*n*)	4
LVEF at hospital admission (%)	32.5 ± 8.1
Preserved RVEF (TAPSE > 20 mm) (*n*)	10
Severe mitral valve insufficiency (*n*)	2
Severe aortic valve stenosis (*n*)	1
SAP (mmHg)	110.00 ± 22.19
DAP (mmHg)	65.10 ± 11.52
MAP (mmHg)	87.55 ± 15.85
PCWP (mmHg)	19.50 ± 12.14
CVD (mmHg)	13.50 ± 6.84
PAM (mmHg)	13.30 ± 7.88
BMI	23.20 ± 1.75
Baseline GFR (at hospital admission)	43 ± 21.60
Need for renal replacement therapy (*n*)	10
Norepinephrine (µg/kg/min)	0.26 ± 0.09
Dobutamine (µg/kg/min)	5.21 ± 1.51
Invasive ventilation (pressure-controlled) (*n*)	10
PEEP (mmHg)	8.10 ± 0.83
*P*_insp_. (mmHg)	20.20 ± 2.44
MVV (l/min)	5.82 ± 0.73
TVI (ml)	471.00 ± 35.50
FiO_2_ (%)	52.40 ± 17.08

*BMI*, body mass index; *CHD*, coronary heart disease; *CVD*, central venous pressure; *DAP*, diastolic arterial pressure; *FiO*_*2*_, fraction of inspired oxygen; *LVEF*, left ventricular ejection fraction; *MAP*, median arterial pressure; *MVV*, minute volume ventilation; *PAM*, mean pulmonary arterial pressure; *PCWP*, pulmonary capillary wedge pressure; *PEEP*, positive end-expiratory pressure; *P insp.*, inspiratory pressure; *RRT*, renal replacement therapy; *SAP*, systolic arterial pressure; *TAPSE*, tricuspid annular posterior systolic elevation; *TVI*, inspiratory tidal volume

The cohort’s mean age was 67 ± 7.6 years, and 80% were male. One-third of the patients had an underlying 3-vessel coronary disease, one-third had a 2-vessel coronary disease, and one-third of the patients had a 1-vessel coronary disease. The coronary-angiography with optimal revascularization, the implantation of VA-ECMO via femoral artery and vein, and the insertion of Impella CP via the contralateral femoral artery were performed in all patients under fluoroscopic control in the cath lab. On admission, the mean systolic ejection fraction was 32.5 ± 8.1%, and the calculated mean glomerular filtration rate (GFR) was reduced to 43 ± 21.6 ml/min. Mean dosages of vasopressors and inotropes during the measurements were 0.26 ± 0.09 µg/kg/min norepinephrine and 5.21 ± 1.51 µg/kg/min dobutamine (Table 1). No significant correlation could be shown between clinical characteristics (e.g., valvular lesions, left ventricular ejection function) and changes of hemodynamics, tissue, and organ perfusion.

Reducing the output of the Impella by a mean of 0.7 l/min and enhancing the output of VA-ECMO by a mean of 0.7 l/min (condition 1) lead to a significant decrease in CI from BL 2.71 ± 0.75 to 2.04 ± 0.64 (*p* = 0.04) and of CO from BL 5.16 ± 1.27 l/min to 3.86 ± 1.11 min (*p* = 0.018) measured by thermodilution. Likewise, the values for CI and CO determined according to the Fick principle also decreased significantly from BL 3.0 ± 0.41 to 2.62 ± 0.34 (*p* = 0.042) and from BL 5.79 ± 0.66 l/min to 5.02 ± 0.571/min (*p* = 0.025), respectively. SvO_2_ simultaneously decreased from BL 60.65 ± 5.64% to 56.44 ± 6.97% (*p* = 0.136) confirming the previous results of hemodynamic measurements. Although the *p*-value did not reach statistical significance due to the small cohort, the numerical decrease seems evident.

**Fig. 2:**
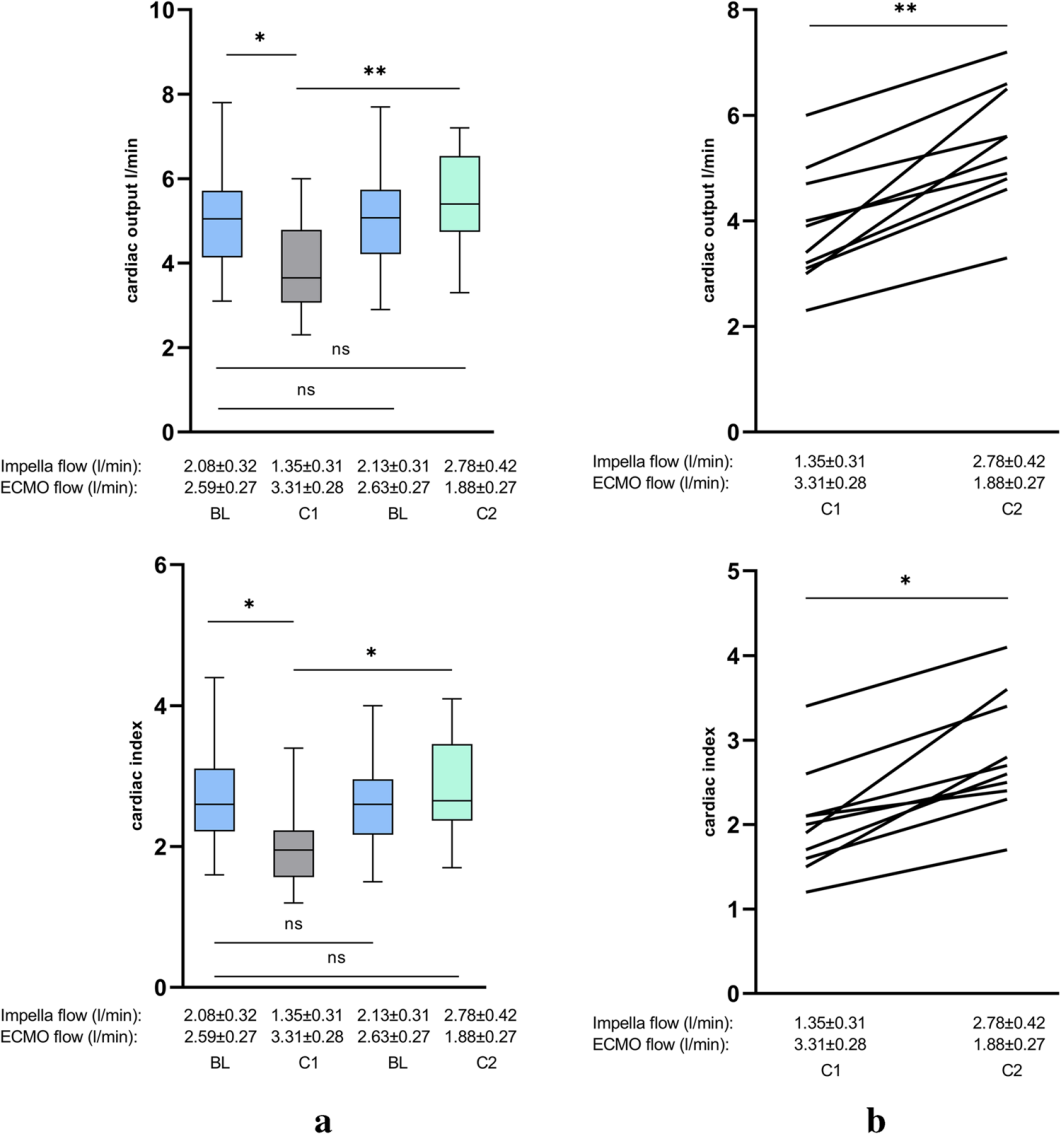
Changes in hemodynamics during Impella and VA-ECMO support by thermodilution. **a** Cardiac output (CO) and cardiac index (CI) during different conditions of Impella and VA-ECMO support. **b** Direct comparison of conditions 1 and 2 during ECMELLA support regarding CI and CO. Comparing condition 1 (VA-EMO > Impella) and condition 2 (Impella > VA-ECMO), condition 2 with enhanced Impella flow (1.35 ± 0.31 l/min to 2.78 ± 0.42 l/min) and reduced VA-ECMO flow (3.31 ± 0.28 l/min to 1.88 ± 0.27 l/min), lead to a significant increase of CO and CI. Between the BL conditions, no significant alterations could be observed (BL, baseline; C1, condition 1; C2, condition 2; ns, not significant; *p* ≧ 0.05, *p < 0.05, **p ≤ 0.01)

**Fig. 3:**
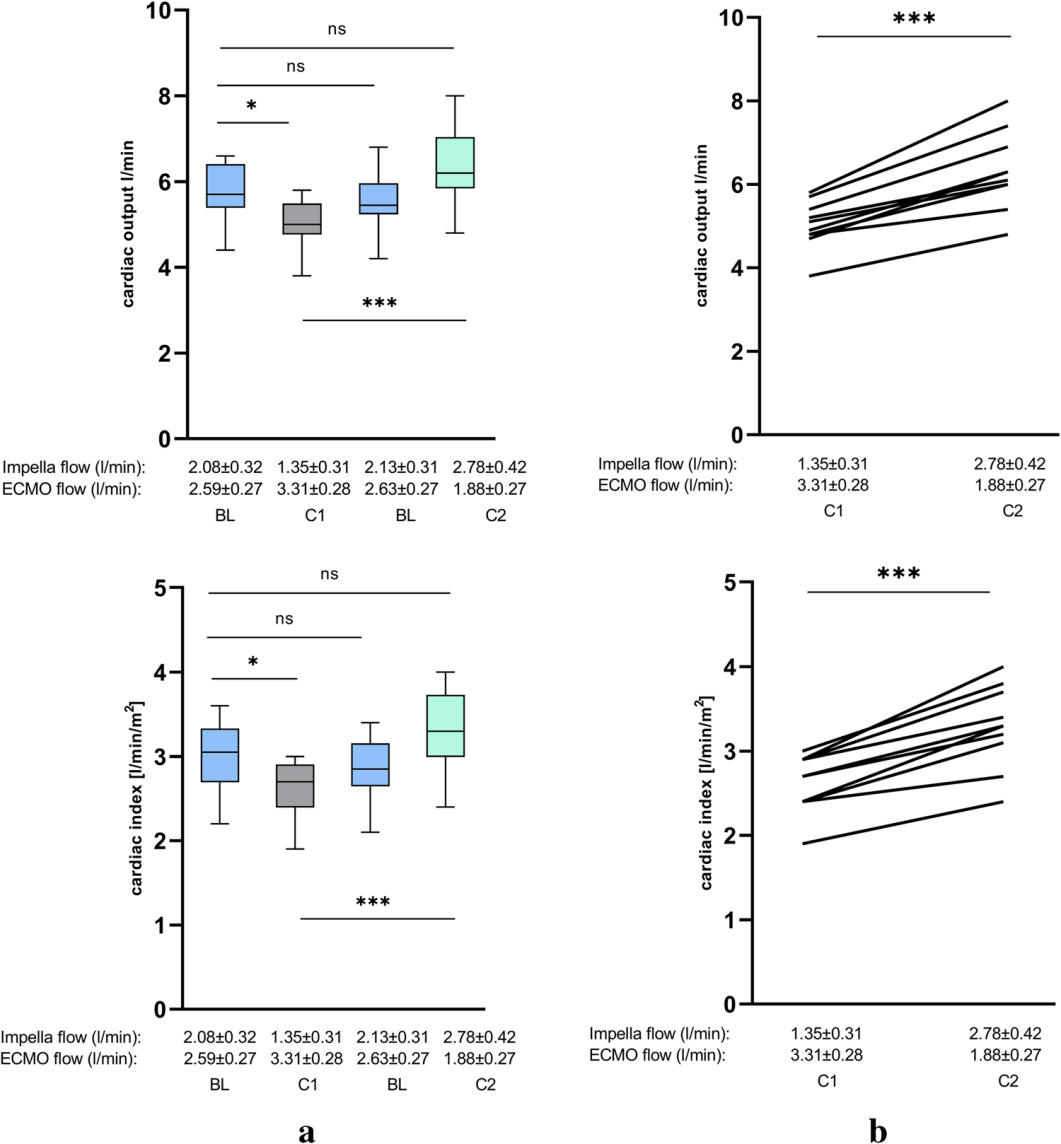
Changes in hemodynamics during Impella and VA-ECMO support by FICK PRINCIPLE. **a** Cardiac output (CO) and cardiac index (CI) during different conditions of Impella and VA-ECMO support. **b** Direct comparison of conditions 1 and 2 during ECMELLA support regarding CI and CO. Comparing condition 1 (VA-EMO > Impella) and condition 2 (Impella > VA-ECMO), condition 2 with enhanced Impella flow (1.35 ± 0.31 l/min to 2.78 ± 0.42 l/min) and reduced VA-ECMO flow (3.31 ± 0.28 l/min to 1.88 ± 0.27 l/min), lead to a significant increase of CO and CI. Between the BL conditions, no significant alterations could be observed (BL, baseline; C1, condition 1; C2, condition 2; ns, not significant; *p* ≧ 0.05, *p < 0.05, **p ≤ 0.01)

**Fig. 4:**
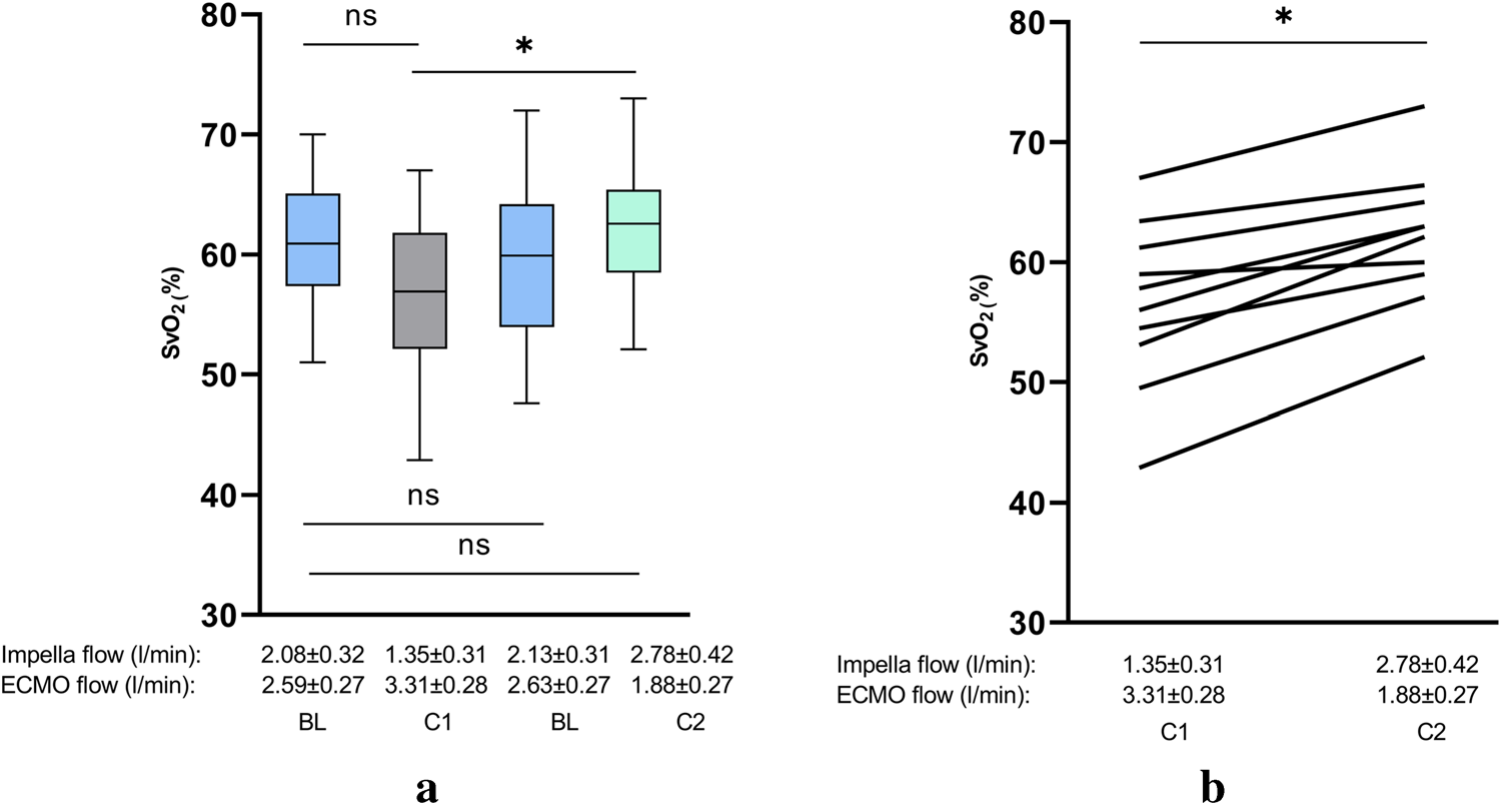
Changes in mixed venous oxygen saturation (SvO_2_) during Impella and VA-ECMO support. **a** SvO_2_ during different conditions of Impella and VA-ECMO support. **b** Direct comparison of conditions 1 and 2 during ECMELLA support regarding SvO_2_. Comparing condition 1 (VA-EMO > Impella) and condition 2 (Impella > VA-ECMO), condition 2 with enhanced Impella flow (1.35 ± 0.31 l/min to 2.78 ± 0.42 l/min) and reduced VA-ECMO flow (3.31 ± 0.28 l/min to 1.88 ± 0.27 l/min), lead to a significant increase of SvO_2_. Between the BL conditions, no significant alterations could be observed (BL, baseline; C1, condition 1; C2, condition 2; ns, not significant; *p* ≧ 0.05, *p < 0.05, **p ≤ 0.01)

**Fig. 5:**
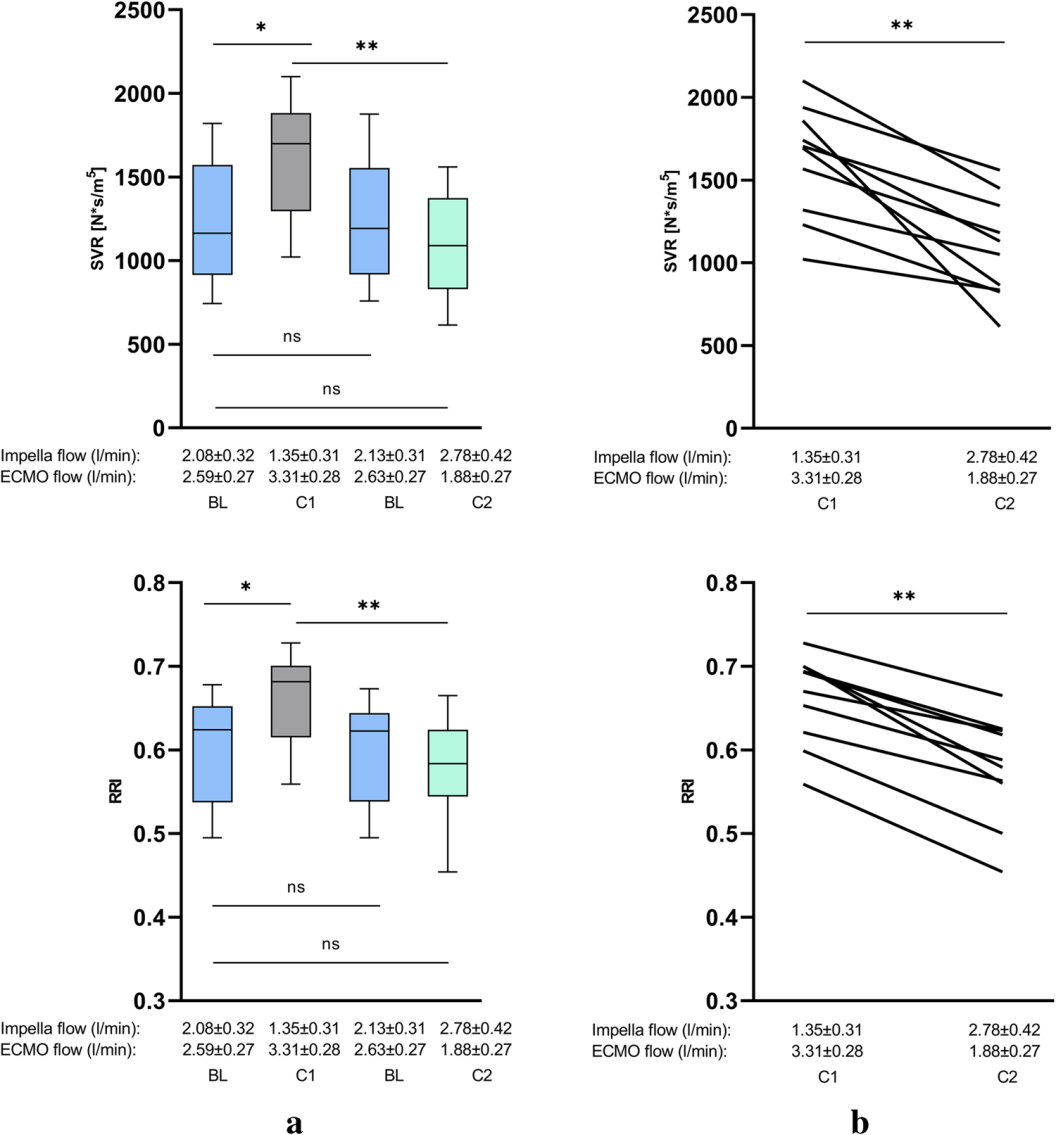
Systemic vascular resistance and renal resistive index and during ECMELLA support. **a** Changes of the renal resistive index (RRI) and of systemic vascular resistance (SVR) during different conditions of Impella and VA-ECMO support. **b** Comparison of conditions 1 and 2 during ECMELLA support. Comparing condition 1 (VA-EMO > Impella) and condition 2 (Impella > VA-ECMO), condition 2 with enhanced Impella flow (1.35 ± 0.31 l/min to 2.78 ± 0.42 l/min) and reduced VA-ECMO flow (3.31 ± 0.28 l/min to 1.88 ± 0.27 l/min), lead to a significant reduction of RRI and SVR. Between the BL conditions, no significant alterations could be observed (BL, baseline; C1, condition 1; C2, condition 2; ns, not significant; *p* ≧ 0.05, *p < 0.05, **p ≤ 0.01)

In addition, the SVR and RRI significantly increase from BL 1235 ± 382 s*cm^−5^ to 1618 ± 337 s*cm^−5^ (*p* = 0.017) and from 0.606 ± 0.07 to 0.652 ± 0.05 (*p* = 0.043) (Table 2, Figs. 2, 3, 4, and 5).

**Table 2:** Parameters of invasive hemodynamic measurement on different levels of biventricular support

	Baseline (BL) *Impella flow* 2.08 ± 0.34 l/min *ECMO flow* 2.59 ± 0.27 l/min	Condition 1 *Impella flow* 1.35 ± 0.31 l/min *ECMO flow* 3.31 ± 0.28 l/min	Baseline (BL) *Impella flow* 2.13 ± 0.31 l/min *ECMO flow* 2.63 ± 0.27 l/min	Condition 2 *Impella flow* 2.78 ± 0.42 l/min *ECMO flow* 1.88 ± 0.27 l/min
CO (l/min) Thermodilution	5.16 ± 1.27	3.86 ± 1.11	5.04 ± 1.14	5.44 ± 1.13
CI Thermodilution	2.71 ± 0.75	2.04 ± 0.64	2.62 ± 0.66	2.85 ± 0.69
SVR (s*cm^−5^) Thermodilution	1235 ± 382	1618 ± 337	1249 ± 400	1086 ± 306
CO (l/min) Fick principle	5.76 ± 0.66	5.02 ± 0.57	5.52 ± 0.68	6.32 ± 0.93
CI Fick principle	3.0 ± 0.41	2.62 ± 0.34	2.87 ± 0.39	3.29 ± 0.47
SvO_2_ (%) DO_2_/VO_2_	60.65 ± 5.64	56.44 ± 6.97	59.82 ± 6.28	62.02 ± 5.64
RRI Doppler ultrasound	0.606 ± 0.07	0.662 ± 0.05	0.603 ± 0.06	0.578 ± 0.06

*CO*, cardiac output; *CI*, cardiac index; *DO*_*2*_, oxygen delivery; *RRI*, renal resistive index; *SVR*, systemic vascular resistance; *SvO*_*2*_, mixed venous oxygen saturation; *VO*_*2*_, oxygen consumption

On the other hand, augmenting the output of the Impella by a mean of 0.7 l/min and reducing the output of VA-ECMO by a mean of 0.7 l/min (condition 2) lead to an increase of CI from BL 2.71 ± 0.75 to 2.85 ± 0.69 (*p* = 0.655) and of CO from BL 5.16 ± 1.27 l/min to 5.44 ± 1.13 l/min (*p* = 0.598) and a reduction of SVR from BL 1235 ± 382 s*cm^−5^ to 1086 ± 306 s*cm^−5^ (*p* = 0.373) measuring by thermodilution. CI and CO measured by Fick principle also increased noteworthy from BL 3.0 ± 0.41 to 3.29 ± 0.47 (*p* = 0.132) and from BL 5.79 ± 0.66 l/min to 6.32 ± 0.93 1/min (*p* = 0.095) respectively. Once again confirming the hemodynamic measurements, SvO_2_ increased from BL 60.65 ± 5.64% to 62.02 ± 5.64% (*p* = 0.608). As previously mentioned, even if the values do not reach statistical significance, there is a clear numerical difference between the measurements again. The RRI dropped from BL 0.606 ± 0.07 to 0.578 ± 0.06 (*p* = 0.299) (Table 2, Figs. 2, 3, 4, and 5). However, *p*-values did not reach statistical significance.

A direct comparison of condition 1 (Impella output 1.35 ± 0.31 l/min, VA-ECMO output 3.31 ± 0.28 l/min) and condition 2 (Impella output 2.78 ± 0.42 l/min, VA-ECMO output 1.88 ± 0.27 l/min) reveals substantial statistically significant alterations of hemodynamics, vascular resistances, RRI, and tissue and organ perfusion. During condition 2, CI values obtained by thermodilution as well as by the Fick principle increased significantly from 2.04 ± 0.64 to 2.85 ± 0.69 (*p* = 0.013) and from 2.62 ± 0.34 to 3.29 ± 0.47 (*p* = 0.001) respectively. Also, both CO values measured by thermodilution as well as by the Fick principle increased in a significant manner from 3.86 ± 1.11 to 5.44 ± 1.13 (*p* = 0.005) and from 5.02 ± 0.57 to 6.32 ± 0.93 (*p* < 0.001). Moreover, SvO_2_ as another parameter that reflects cardiac output increased significantly from 56.44 ± 6.97% to 62.02 ± 5.64% (*p* = 0.049) whereas SVR and RRI significantly decreased from 1618 ± 337 s*cm^−5^ to 1086 ± 306 s*cm^−5^ (*p* = 0.002) and from 0.662 ± 0.05 to 0.578 ± 0.06 (*p* = 0.003) (Figs. 2, 3, 4, and 5).

Taken together, all methods of hemodynamic measurement used here showed comparable findings regarding the CO, CI, SVR, and RRI during all conditions of hemodynamic support. For further validation, the SvO_2_ could be documented as another independent parameter reflecting the “real,” meaning overall CO.

## Discussion

Here we demonstrate that PA catheterization for hemodynamic monitoring is applicable and helpful to optimize the management of patients in CS with biventricular MCS. In detail, the ratio of Impella flow > ECMO flow is superior to VA-ECMO > Impella flow resulting in a more profound increase in cardiac output (CO) with subsequent improvement of organ perfusion.

These findings seem particularly relevant since in everyday clinical practice the adequate selection, differentiated use, and the most optimal flow balance of MCS continue to be controversially discussed. Nevertheless, the management and control of MCS devices during therapy in CS are often operator-based or follow institutional policy. While VA-ECMO provides both hemodynamic and respiratory support, its retrograde blood flow increases afterload, causing further deterioration in myocardial function [27]. Moreover, surged cardiac afterload elevates myocardial oxygen demand and myocardial wall stress deteriorating myocardial microcirculation, and hampering myocardial recovery. Recent evidence from Napur and colleagues however demonstrated protective effects of Impella support on myocardial regeneration which may be subjected to its antegrade blood flow and its subsequent ventricular unloading [28]. Nevertheless, the Impella lacks any respiratory support.

Current recommendations differentiate the use of different MCS types depending on the estimated underlying clinical problems in CS (e.g., right vs. left ventricular insufficiency, concomitant respiratory failure) but do not adequately address secondary effects such as concomitant left ventricular dilation and failure during VA-ECMO therapy [29].

Therefore, the ECMELLA strategy has been progressively applied combining the unloading effects of the left ventricle and the reduction of afterload [3, 5]. ECMELLA therapy is further associated with lower mortality in CS compared to MCS only with VA-ECMO [3, 5, 6].

However, the underlying pathophysiological and mechanistic effects of device interaction during ECMELLA therapy are often poorly understood. Therefore, adequate hemodynamic monitoring for therapy control is warranted. Even though echocardiography is easily applicable and non-invasive, its use during Impella support is limited as the position of the device in the left ventricle may impair the calculation of hemodynamic data. Moreover, the calculation of hemodynamic parameters based on Doppler signals relies completely on flow alterations. Thus, invasive monitoring of hemodynamics should be considered. However, the canonical understanding of hemodynamics often focusses exclusively on the “native” CO, without taking into account the “real” CO, generated by the interaction of the devices (opposing laminar blood flows, different flow levels) and the myocardium.

As a consequence, the discussion on the limitations of applicable options for hemodynamic monitoring during biventricular support continues according to the basic understanding. In detail, regarding the “native” CO, the accuracy of the thermodilution measurement has to be seriously questioned during VA-ECMO therapy since the “native” CO will be underestimated during VA-ECMO therapy but overestimated in veno-venous ECMO (VV-ECMO) due to additional venous recirculation. Thus, the measurement of the “native” CO by thermodilution method during biventricular support is not reliable. According to our data, however, a differential evaluation should be considered for the “real” CO output that is generated by the entire patient and machine circuit. In order to prove this argumentation, further measurement techniques were carried out and limitations will be discussed.

The Fick principle was actually developed for spontaneously breathing people without any shunt circuit as we have in MCS. However, when VA-ECMO support is required in cardiogenic shock, the vast majority of patients are mechanically ventilated and VA-ECMO circulation occurs in parallel to native circulation. During our measurements, the FiO_2_ of the respirator and the oxygen concentration of VA-ECMO remained stable. Under these conditions, the additionally measured mixed venous oxygen saturation (SvO2) as another independent parameter correlates with the CO during different flow levels of VA-ECMO and Impella support, confirming the accuracy of the Fick principle even during biventricular support. Contrary to expectations, particularly SvO_2_ does not correlate with the increase of VA-ECMO flow. However, Fick’s principle can only be used for hemodynamic measurement with the limitation that blood is taken from the right radial artery.

Pulse pressure contour analysis (e.g., PiCCO®) is limited by the fact that MCS devices like VA-ECMO and Impella provide a laminar blood flow. In addition, estimating CO using Doppler ultrasound sonography can lead to inaccurate data representation in patients with VA-ECMO and Impella, as the Impella creates artificial sonographic artifacts. To achieve a reliable velocity time integral (VTI) is difficult. These measurement techniques are therefore rather unsuitable for therapy control during biventricular support.

Since we were able to show that the results of our measurements (CO and CI including SvO2) were fundamentally consistent across all used methods, we also determined the RRI, another parameter for CO-independent representation of vascular resistance, to further validate the data of systemic vascular resistance (SVR). Again, comparable values resulted.

In summary, we think that our data allow important insights concerning the most optimal therapy control during biventricular support. Furthermore, our results also agree with those of Ohira et al., who also described an increase of CO when increasing Impella support in the case of biventricular MCS [9].

Ultimately it can be concluded, that the present findings are consistent with the notion that in the absence of alternative gold-standard techniques, invasive hemodynamic monitoring by PA catheterization should be considered to visualize fundamental hemodynamic changes of the “real” CO in patients during biventricular MCS. Thus, invasive hemodynamic monitoring can be used to navigate both devices toward an optimized use. The evaluation of the “native” CO which depends on improving myocardial contractility however becomes particularly interesting again during the weaning phase of devices.

Nevertheless, our results have limitations. One is the small number of patients and the experience of a single center that limits the interpretation of the data. However, basic findings from this first case series to investigate the specific impact of Impella and VA-ECMO on the “patient-and-machine” hemodynamic system, including vascular resistance, and tissue- and organ perfusion are promising but require further evaluation in larger scale trials. Moreover, clinical outcomes of patients should be integrated when investigating the device settings in ECMELLA patients and should be part of further studies.

## Data Availability

Data will be made available at individual request.
